# Severe Malaria Not Responsive to Artemisinin Derivatives in Man Returning from Angola to Vietnam

**DOI:** 10.3201/eid2007.140155

**Published:** 2014-07

**Authors:** Nguyen Van Hong, Alfred Amambua-Ngwa, Nguyen Quang Tuan, Do Duy Cuong, Nguyen Thi Huong Giang, Nguyen Van Dung, Ta Thi Tinh, Nguyen Van Tien, Bui Quang Phuc, Tran Thanh Duong, Anna Rosanas-Urgell, Umberto D’Alessandro, Jean-Pierre Van Geertruyden, Annette Erhart

**Affiliations:** National Institute of Malariology, Parasitology and Entomology, Hanoi, Vietnam (N. Van Hong, T.T. Tinh, B.Q. Phuc, T.T. Duong);; Medical Research Unit, Fajara. The Gambia (A. Amambua-Ngwa, U. D’Allesandro);; Bach Mai Hospital, Hanoi (N.Q. Tuan, D.D. Cuong, N.T.H. Giang, N. Van Dung, N. Van Tien);; Institute of Tropical Medicine, Antwerp, Belgium (A. Rosanas-Urgell, A. Erhart);; University of Antwerp, Antwerp (J.-P. Van Geertruyden)

**Keywords:** malaria, severe malaria, cerebral malaria, parasites, Plasmodium falciparum, severe malaria, drug resistance, artemisinins, travel medicine, Vietnam, Angola

## Abstract

Resistance to artemisinin derivatives, the most potent antimalarial drugs currently used, has emerged in Southeast Asia and threatens to spread to Africa. We report a case of malaria in a man who returned to Vietnam after 3 years in Angola that did not respond to intravenous artesunate and clindamycin or an oral artemisinin-based combination.

Artemisinin derivatives are used in combination with other drugs for treatment of *Plasmodium falciparum* malaria. Nevertheless, *P. falciparum* resistance to artemisinins has been recently detected in 4 countries (Cambodia, Thailand, Myanmar, and Vietnam) in the Greater Mekong subregion in Southeast Asia (*1*). Artemisinin resistance could spread from these countries to other regions, including sub-Saharan Africa, where the incidence of malaria is highest, and where artemisinin resistance would have devastating consequences. Increased and uncontrolled travel between Asia and Africa might contribute to the spread of artemisinin-resistant malaria parasites.

An estimated 40,000 Vietnamese workers travel annually to Angola (*2*), where *P. falciparum* malaria is the leading cause of illness and death (*3*,*4*). Over the past year, an increasing number of severe malaria cases have been identified among Vietnamese migrants returning from Angola (*5*). During March–mid-April 2013, five deaths from malaria among Vietnamese workers in Angola were reported (*6*–*8*). We report a case of malaria in a Vietnamese man who returned from Angola and did not respond to intravenous artesunate or oral artemisinin-based combination therapy.

## The Study

In April 2013, a 58-year-old Vietnamese man was admitted to Bach Mai Hospital in Hanoi, Vietnam, because of high fever, jaundice, and lack of consciousness. Eleven days before hospitalization, he had returned from Saurimo City in Angola, where he had been working in the construction industry for the past 3 years, to his home village in malaria-free Nam Dinh Province, 80 km from Hanoi. Four days after returning from Angola, he reported fatigue, fever with chills, and a cough. Two days later, he went to the local district hospital where he was given a diagnosis of bronchitis and received a third-generation cephalosporin (cefixime) and antipyretics for 3 days. Ten days after his arrival in Vietnam, the patient came to the emergency department of Bach Mai Hospital because of continuous high fever (body temperature 39°C–40°C), dyspnea, and urinary incontinence. He was given fosfomycin for pyelonephritis.

Because his clinical condition worsened rapidly, the patient was transferred the next day to the Infectious Diseases Department in the same hospital because cerebral malaria was diagnosed. At admission to the department, the patient (weight 58 kg) was in a confused state and had a Glasgow coma score of 13/15, generalized convulsions, jaundice, and tachypnea (respiration rate 25 breaths/min), a body temperature of 38.5°C, a blood pressure of 130/80 mm Hg, and pulse rate of 121 beats/min. Clinical examination detected hepatomegaly and cracklings in both lungs. Blood tests showed the following results: leukocyte count 13.3 × 10^9^/cells/L, hemoglobin 153 g/L, platelet count 20.9 × 10^9^/L, blood urea nitrogen 19.8 mmol/L, serum creatinine 135 mmol/L, aspartate aminotransferase 125 IU/L, alanine aminotransferase 45 IU/L, C-reactive protein 16 mg/L, procalcitonin >120 ng/mL; total bilirubin 116 µmol/L; direct bilirubin 11.5 µmol/L; and standard levels of electrolytes.

Parasite density/microliter of blood was calculated after counting the total number of *P. falciparum* trophozoites/200 leukocytes and assuming a leukocyte concentration of 8,000 cells/μL. Microscopy identified *P. falciparum* trophozoites at a concentration of 378,470/µL and some schizonts. A chest radiograph showed bilateral pneumonia. Analysis of cerebrospinal fluid and a computed tomographic scan of the brain showed standard results. Tests results for HIV and hepatitis B surface antigen were negative.

**Figure 1:**
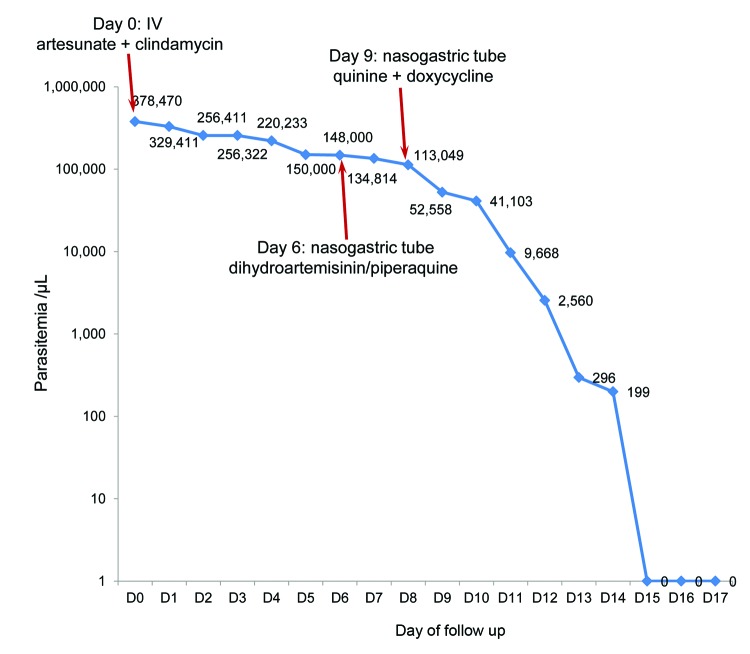
Evolution of *Plasmodium falciparum* parasite density (log scale) by day after start of antimalaria treatments for man with severe malaria who returned from Angola to Vietnam in April 2013. Values are parasites/microliter of blood. IV, intravenous.

The patient was given a diagnosis of *P. falciparum* cerebral malaria and treated with intravenous (IV) artesunate (batch no. 511004; Pharbaco, Hanoi, Vietnam) (60 mg every 12 h, loading dose 120 mg at admission) and IV clindamycin (600 mg every 12 h). After 24 h of hospitalization, the patient had a Glasgow coma score of 11, a body temperature of 39.7°C, and a parasite density of 329,411 parasites/µL (Figure 1), a blood pressure of 70/40 mm Hg, and a respiration rate of 30 breaths/min. An ultrasound examination showed hepatomegaly and bilateral pleural effusion. At day 2 of hospitalization, the patient was treated with intubation and mechanical ventilation. At day 4, the hemoglobin level had decreased to 9.3 g/dL and the patient was given a blood transfusion. After 5 consecutive days of treatment with IV artesunate and clindamycin, the patient remained comatose and had continuous fever (39°C) and high parasite density (148,000 parasites/µL).

At day 6 of hospitalization, artesunate and clindamycin were discontinued, and oral treatment with dihydroartemisinin (40 mg/tablet)/piperaquine (320 mg/tablet) was administered through a nasogastric tube for 3 days (4 tablets/day the first day, then 2 tablets/day). After 3 days of treatment, the patient was still febrile (38.5°C) and had a Glasgow coma score of 12 and a parasite density of 113,409 parasites/µL (Figure 1). Quinine (1,750 mg/day) and doxycycline (200 mg/day) were then administered at day 9 through a nasogastric tube (injectable quinine was not available). Twelve hours after the first dose of quinine, parasite density was 55,558 parasites/µL. It decreased to 9,668 parasites/µL 72 h at day 11 after starting quinine treatment, when fever eventually subsided. Two days later, the patient regained consciousness, and at day 15 of hospitalization, blood slides were negative for parasites (Figure 1). The patient eventually recovered and was discharged 35 days after admission.

**Figure 2:**
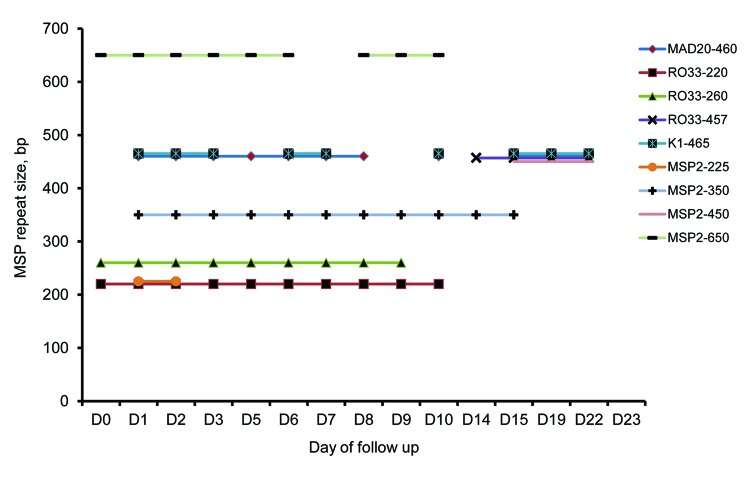
Clonal complexity of *Plasmodium falciparum* strain that caused severe malaria in man who returned from Angola to Vietnam after treatment, as determined by merozoite surface protein 1 (MSP1) and MSP2 repeat length molecular typing. Allele sizes were detected by capillary electrophoresis of amplified MSP repeat regions and shown for each follow-up sample.

Species-specific PCR (*9*) of a blood sample obtained on day 3 of hospitalization confirmed the diagnosis of *P. falciparum* monoinfection. Further genotyping by using nested PCR (merozoite surface proteins 1 and 2 repeat markers) and capillary electrophoresis (*10*) of several filter paper blood spots obtained during the 23 days of hospitalization confirmed that the patient had a polyclonal *P. falciparum* infection with ≥2 clones. These clones might have persisted from admission through day 10 of hospitalization (Figure 2).

The quality of IV artesunate used was determined by using high-performance liquid chromatography according to USP34 NF29 specifications at the National Institute of Drug Quality Control (Hanoi, Vietnam) (http://nidqc.org.vn/). The drug was found to be acceptable.

## Conclusions

This clinical case of suspected artemisinin resistance originating from sub-Saharan Africa is of concern because the patient had probably been infected in Angola and because the clinical presentation differed from delayed parasite clearance reported in Southeast Asia (*1*). The patient had never had malaria and had worked continuously in Angola for the past 3 years before his return (direct flight to Hanoi and then directly by car to his home village) to a malaria-free area in Vietnam. Given the onset of symptoms only 4 days after his return, the patient was most likely infected in Luanda Sul Province, Angola, to which malaria is hyperendemic and where Vietnamese workers have reportedly died of malaria (*6*–*8*).

Five days of treatment with intravenous artesunate and clindamycin and a 3-day regimen of dihydroartemisinin/piperaquine did not clear the infection because parasite density remained high (>100,000 parasites/µL) until the eighth day of hospitalization. Parasite density showed a logarithmic decrease only after treatment was changed to quinine and doxycycline.

An external quality control of all blood slides was performed by an expert microscopist at the National Institute of Malariology, Parasitology and Entomology in Hanoi; the parasite densities were confirmed. Therefore, this case is suggestive of *P. falciparum* tolerance to artemisinin derivatives. However, because no blood samples for pharmacokinetic or in vitro studies were obtained, drug resistance could not be confirmed.

Identification of *P. falciparum* malaria parasites tolerant to arteminins raises serious concerns that artemisin-resistant strains have emerged or spread in Africa. This finding would be a major public health disaster and needs to be urgently confirmed by larger treatment efficacy studies in Angola.

## References

[1] World Health Organization. Joint assessment of the response to artemisinin resistance in the greater Mekong sub-region. November 2011–February 2012. Summary report [cited 2013 Dec 11]. http://whothailand.healthrepository.org/bitstream/123456789/1484/3/120418-Artemisinin-Resistance-in-GMS-SummaryReport.pdf

[2] Dan Tri (official Journal of the Vietnamese government), 2013. Vietnamese workers are tempted by high income in Angola. Intellectuals Magazine. Interview with Prime Minister Le Thanh Hoa, Deputy Minister of Labour, Invalid and Social Vietnam [cited 2013 Sep 22]. http://dantri.com.vn/xa-hoi/lao-dong-viet-bi-cam-do-boi-muc-thu-nhap-cao-o-angola-733732.htm.

[3] Demographic and Health Surveys. Angola malaria indicators, survey 2011 [cited 2013 Nov 26]. http://www.measuredhs.com/what-we-do/survey/survey-display-395.cfm

[4] US Agency for International Development. President’s Malaria Initiative. (PMI), Country Profile, Angola, April 2013 [cited 2014 Mar 28]. http://www.pmi.gov/countries/profiles/angola_profile.pdf

[5] Health and Life. Vietnam Ministry of Health. Imported malaria warning, 2013 [cited 2013 Dec 1]. http://suckhoedoisong.vn/y-te/canh-bao-sot-ret-ngoai-nhap-khang-thuoc-20130814091119352.htm

[6] Institute of Malariology. Parasitology and Entomology, Quy Nhon, Vietnam, 2013. What can be seen from Vietnam workers who died of malaria in Angola? [cited 2013 Apr 18]. http://www.impe-qn.org.vn/impe-qn/vn/portal/InfoDetail.jsp?area=58&cat=944&ID=6382

[7] Dan Tri (official journal of the Vietnamese government). A Vietnamese worker died of complicated malaria in Angola, 2013 [cited 2013 Aug 14]. http://dantri.com.vn/xa-hoi/them-mot-lao-dong-viet-tu-vong-vi-sot-ret-ac-tinh-o-angola-742723.htm

[8] One more Vietnamese male worker died of malaria in Angola. Education Magazine, 2013 [cited 2013 Nov 30]. http://giaoduc.net.vn/Xa-hoi/Phat-hien-them-mot-nam-lao-dong-chet-vi-sot-ret-tai-Angola/299241.gd

[9] Rubio JM, Post RJ, van Leeuwen WM, Henry MC, Lindergard G, Hommel M. Alternative polymerase chain reaction method to identify *Plasmodium* species in human blood samples: the semi-nested multiplex malaria PCR (SnM-PCR). Trans R Soc Trop Med Hyg. 2002;96(Suppl 1):S199–204. 10.1016/S0035-9203(02)90077-5 12055839

[10] Liljander A, Wiklund L, Falk N, Kweku M, Martensson A, Felger I, Optimization and validation of multi-coloured capillary electrophoresis for genotying of *Plasmodium falciparum* merozoite surface proteins (msp1 and 2). Malar J. 2009;8:78. 10.1186/1475-2875-8-78 19386138 PMC2680902

